# Gap Junction-Dependent and -Independent Functions of Connexin43 in Biology

**DOI:** 10.3390/biology11020283

**Published:** 2022-02-11

**Authors:** Yi Zhu

**Affiliations:** Children’s Nutrition Research Center, Department of Pediatrics, Baylor College of Medicine, Houston, TX 77030, USA; yi.zhu@bcm.edu

**Keywords:** GJA1, Connexin43, gap junction, channel, hemichannel, Cx43-20k

## Abstract

**Simple Summary:**

Connexin43 is one of the 21 members of a large protein family that forms intercellular gap junction complexes. It plays a critical role in development by allowing two adjacent cells to exchange cellular content. Mouse deletion studies have demonstrated its critical roles in many organs, including cardiac muscle, blood vessels, bone, adipose tissue and more. However, as the most expressed and most studied connexin in the family, Connexin43 surprisingly shows many gap-junction-independent roles, spanning from the forming of hemichannels, to regulating membrane trafficking, to regulating mitochondrial function. Connexin43 is also unique in the family by forming many smaller polypeptides through alternative utilization of its mRNA molecule or cleavage of the translated full-length protein. This review summarizes recent advances on Connexin43’s gap-junction-dependent and -independent functions in physiology. The knowledge will expand our understanding of how a gene grows its function by multiplexing its mRNA and protein. In the end, this may also guide us to develop Connexin43-based therapy for metabolic conditions, cancer, and other related diseases.

**Abstract:**

For the first time in animal evolution, the emergence of gap junctions allowed direct exchanges of cellular substances for communication between two cells. Innexin proteins constituted primordial gap junctions until the connexin protein emerged in deuterostomes and took over the gap junction function. After hundreds of millions of years of gene duplication, the connexin gene family now comprises 21 members in the human genome. Notably, *GJA1*, which encodes the Connexin43 protein, is one of the most widely expressed and commonly studied connexin genes. The loss of *Gja1* in mice leads to swelling and a blockage of the right ventricular outflow tract and death of the embryos at birth, suggesting a vital role of Connexin43 gap junction in heart development. Since then, the importance of Connexin43-mediated gap junction function has been constantly expanded to other types of cells. Other than forming gap junctions, Connexin43 can also form hemichannels to release or uptake small molecules from the environment or even mediate many physiological processes in a gap junction-independent manner on plasma membranes. Surprisingly, Connexin43 also localizes to mitochondria in the cell, playing important roles in mitochondrial potassium import and respiration. At the molecular level, Connexin43 mRNA and protein are processed with very distinct mechanisms to yield carboxyl-terminal fragments with different sizes, which have their unique subcellular localization and distinct biological activities. Due to many exciting advancements in Connexin43 research, this review aims to start with a brief introduction of Connexin43 and then focuses on updating our knowledge of its gap junction-independent functions.

## 1. The Emergence of Connexin43 as a Gap Junction Protein in Evolution

A gap junction is a specialized intercellular channel, formed by docking two hemichannels from each cell [1,2]. For the first time, gap junction proteins permitted direct cell–cell transfer of cytoplasmic contents, representing a hallmark in evolution, from single-cell organisms to multicellular organisms, by allowing the whole being to function in a manner that was greater than the sum of each individual part. One would imagine that the genes for such an important cellular feature as gap junction would be highly conserved. Paradoxically, gap junction proteins were initially coded by innexin family genes; connexin genes took over the gap junction function for the first time in the deuterostomes [3]. Innexin- and connexin-encoded proteins all possess 4-transmembrane domains, but they share no sequence similarity. This connexin–innexin dichotomy suggests a convergent solution to the need for intercellular communication [3]. After the connexin proteins took over the gap junction function, innexins evolved into pannexins, which are specialized in hemichannel function [4]. The inability of pannexins to form a full intercellular junction channel has been attributed to their extensive glycosylation, which physically hinders the interaction of two hemichannels [5].

Through a series of gene duplications, the mammalian connexin gene family now comprises about 20 members, the exact number depending on individual species [6]. Based on similarity in DNA sequences, connexin genes have been classified into several classes, including α, β, γ, and δ-connexins, named using the prefix “GJ” for “gap junction” (e.g., GJA1 for the first member of the α class). Their protein products are named according to predicted protein molecular mass, calculated in kilodaltons (kD), and a prefix “CX” [7]. Gap junctions and connexins have been extensively studied in the past century, from the observation of the gap junction structure in the 1960s [1,8], to the structural and functional characterization of individual family members [9], to using genetic models in various cell populations to understand their physiological role in vivo. Discoveries of connexin’s functions in various cell types are still ongoing. The Human Genome Project, large-scale cohort studies, and bioinformatic analysis of the genome and phenotype/disease association have revealed several diseases linked to mutations in connexin genes [10,11]. Many reviews have covered various aspects of the gap junction and connexins, i.e., their structure [12], their synthesis and trafficking [13,14], their regulation and functions [15], and gap junction-modulating molecules as potential therapeutics [16,17], among more comprehensive overviews [18,19,20]. 

Discovered using northern blot analysis of rat heart lysates, as a homolog to liver gap junction gene Connexin32 in 1987 [21], Connexin43 (Cx43) is now known to be widely expressed in mammalian tissues and is one of the most studied connexin proteins in the family. Many intracellular molecules, such as inorganic salts, sugars, amino acids, and nucleotides can pass through Cx43 gap junction [22]. Within the limits of its scope, this review focuses on Cx43, mostly in the metabolic organs. Due to the structural similarity of connexin proteins, Cx43 studies offer valuable information for studies of other connexins; however, Cx43 is also unique in comparison to other connexins due to the multitude of its gap junction-dependent and -independent roles in several cell types, which is the focus of this review.

## 2. Synthesis of Connexin43 and Forming of Gap Junctions on the Plasma Membrane 

The Cx43 protein possesses four transmembrane domains, two extracellular loops, one intracellular loop, and N-terminal and C-terminal tails that are both intracellular [12]. They assemble into hexamers on the plasma membrane to carry out the protein’s functions [12]. Like most trans-membrane proteins in eukaryotes, Cx43 is synthesized by ribosomes that are bound to the endoplasmic reticulum (ER) membrane. The nascent Cx43 peptide is recognized by a signal recognition particle (SRP) to facilitate the translocation of the SRP/ribosome/nascent-polypeptide-chain/mRNA complex to a channel in the ER membrane [23,24,25]. The nascent peptide is initially confined to the hydrophilic lumen of the channel, before integration into the hydrophobic ER membrane environment [26]; it is then trafficked to the plasma membrane via the canonical ER-Golgi trafficking mechanism [27,28]. The final insertion of gap junction into the plasma membrane is achieved by fusion of Cx43-bearing cytoplasmic vesicles with the plasma membrane [29]. 

The place of Cx43 connexon assembly is likely in the trans-Golgi network (TGN) [30]. Evidence in a cell-free system demonstrated the full assembly of gap junction connexons, composed of Cx43 in microsomes, suggesting that the oligomerization of Cx43 is driven by its biophysical properties and implying that ER membranes take the place of connexon assembly in live cells [31,32]. However, using cultured cells, it was demonstrated around the same time that Cx43 connexon assembly occurs after exiting the ER, probably in the TGN [33]. This model was later supported by genetically engineered connexins that encode ER-retention signals, maintained as an apparent monomer [34]. This discrepancy between the cell-free model and cultured cells may be reconciled by the discovery of an ER-localized, 29-kDa thioredoxin-family protein that inhibits the oligomerization of Cx43 in the ER [35]. Finally, along the secretory pathway, Cx43 is sorted from the ER to the plasma membrane, regulated by multiple proteins, such as 14-3-3 proteins [36,37,38] and caveolin [39,40].

Cx43 is phosphorylated extensively on its C-terminal tail soon after synthesis, and its phosphorylation state changes as it traffics through the ER and Golgi to the plasma membrane, forming into a gap junction structure. Notably, MAPK, PKC, and Src can phosphorylate Cx43 at multiple sites on its C-terminal tail to regulate its trafficking to the plasma membrane, formation of gap junctions, and degradation through endocytosis, which has been reviewed previously [41].

Once delivered to the plasma membrane and assembled into hexamers, Cx43 connexons immediately function as hemichannels, playing important roles in physiology and pathology, which has been extensively reviewed in the literature [42,43,44]. The estimated effective pore size of the Cx43 connexon falls within a range of 10–16 Å; the exact pore sizes by 3D structural measurements under closed or open states are not yet available for the Cx43 connexon [45,46]. Studies in HeLa cells showed that the Cx43 hemichannel gating properties resembled those of the corresponding Cx43 cell–cell full gap junction channels. A non-selective channel would have an inverse linear correlation to the length of the channel. The conductance of fully open single hemichannels is ≈220 pS, approximately twice the Cx43 cell–cell channel conductance, which agrees with the conductance property of a non-selective channel [47]. In fact, the Cx43 connexon shows weak ionic selectivity, likely regulated by the electrostatic interactions [48]. 

The Cx43 hemichannel opening was induced at potentials greater than +60 mV [47]. Given that the resting membrane potential of most cells is negative (for example, −70 mV for neurons and −8.4 mV for erythrocytes [49]), the open probabilities of the Cx43 hemichannel are estimated to be very low under physiological conditions [47]; this agrees with the notion that their constant opening would result in membrane depolarization and the depletion of small molecules from the cytoplasm. In the absence of overt stimulation, HeLa cells uptake ethidium bromide at a low basal rate, suggesting the existence of the infrequent opening of hemichannels or importing ethidium bromide by HeLa cells through alternative mechanisms [47]. Curiously, connexon activation might be a common response to metabolic inhibition [50,51,52]. The Cx43 hemichannel has been implicated in paracrine function via transporting various nucleotides. The most well-known nucleotide that passes through Cx43 hemichannels is ATP, which activates several purinergic receptors on nearby cells and plays important roles in inflammation [53]. 

Contact between two oocytes immediately promotes the directed movement of connexin to the appositional region of the cells [54]. Opposed Cx43 connexons from two cells form a complete gap junction. A few Cx43 gap junctions oligomerize to form “seeding plaques” for the clustering of the same type of connexons [55], other connexons [56], and even other proteins [57], to form an array of connexons. This gap junction plaque was initially visualized by electron micrograph before the connexin proteins were discovered [1]. These channels allow the exchange of nutrients, metabolites, ions, and small molecules of up to ~1 kDa between cells. The gating of the Cx43 channel has been extensively studied, including extracellular Ca^2+^ [58] and intracellular pH [59,60], with several molecular models proposed, focusing on the role of the C-terminal tail of the connexin protein [61,62]. In contrast to the Cx43 hemichannel, evidence suggests that, under a wide range of intracellular conditions, most gap junction channels are gated open more often than closed [22]. 

The half-life of Cx43 is surprisingly short. Several cell culture studies have estimated the half-life of Cx43 to be just a few hours [63,64]. In isolated perfused adult rat hearts, Cx43 turnover kinetics showed a mono-exponential decay curve, with a calculated half-life of 1.3 h [65]. Proteasomal and lysosomal pathways [66,67] and autophagy-related pathways [68] are two major mechanisms of Cx43 degradation.

## 3. Connexin43 Gap Junction and Hemichannel Function in Physiology 

Cx43-mediated cell-to-cell communication plays a vital function in development. The deletion of Cx43 in mice leads to perinatal mortality, due to cardiac malformation and the obstruction of the right ventricular outflow tract of the heart [69]. No complete loss of function of Cx43 has been identified in humans. However, point mutations that compromise its gap junction activity have been found in patients with heart malformations [70,71] and oculodentodigital dysplasia [72,73].

The development of floxed mice 20 years ago [74] allowed cell type-specific elimination of the Cx43 protein in different cell types, sometimes in an inducible fashion. Since then, a huge body of literature has demonstrated the critical functions of Cx43 in various tissues and cells in mice. Table 1 summarizes the major phenotypes of Cx43 deletion in metabolic organs, including heart, vasculature, hepatocytes, adipose tissue, and bone, highlighting an important role for Cx43 in metabolism.

While many studies focus on the gap junction function of Cx43, plasma membrane-inserted Cx43 hexamer immediately function as hemichannels, before docking with another cell’s hemichannel to form a gap junction [75]. Some Cx43 expressing cells, such as immune cells, travel in the circulation or migrate in the tissue and, thus, do not maintain stable contact with other cells. So Cx43 in those cells is expected to mainly function as hemichannels, providing a mechanism for cells to communicate with their environment or nearby cells. Substances released via the Cx43 hemichannels include ATP [76,77], glutamate [78], prostaglandins [79], or metabolic substances like NAD+ [80] or glutathione [81]. These molecules can then act on nearby cells, eliciting an important paracrine signaling cascade. The interplay of Cx43 hemichannels with intercellular signaling opens a new pathway to coordinate cellular events [82]. For example, Cx43 hemichannel-mediated ATP release plays a vital role in the inflammatory response to foreign body [77] and macrophage activation during sepsis [83]. Astrocytic Cx43 hemichannels control the release of small molecules, such as ATP and glutamate, into the extracellular space, to coordinate and maintain physiologic brain activity [78]. Secreted small molecules can also act on the cell itself, playing an autocrine function. One example is that the Cx43 hemichannel induces cancer cell migration, through the release of adenosine nucleotide/nucleoside, which subsequently engages the adenosine receptor 1 (ADORA1) and AKT signaling on the same cell to sever as the migration lead [84].

**Table 1:** The genetic manipulation of Cx43 in different cell types reveals its physiological functions in corresponding tissues. The “Cell Type” column shows in which cell type(s) Cx43 was deleted. The “Promoter” column shows which promoter was used to drive the CRE expression. The “Major Phenotype” column shows the most relevant phenotype in the corresponding mouse model, with references given in the “References” column.

Cell Type	Promoter	Major Phenotype	References
Cardiomyocytes	*Myh6*	Slow conduction and sudden arrhythmic death	[85]
Endothelial cells	*Tek*	Hypotension and bradycardia in mice	[74]
Smooth muscle	*Myh11*	Defective in remodeling processes in response to vascular injury	[86]
Thermogenic adipocytes	*Ucp1*	Impaired cold-induced adipose tissue beiging	[87]
Hepatocytes	*Alb*	Impaired glucose tolerance under high fat-diet feeding	[88]
Cardiac macrophage	*Cx3cr1*	Delay in atrioventricular conduction	[89]
Bone cells (osteoblasts or osteocytes)	*Twist2*, *Col1a1*, *Bglap*, *Dmp1*	Phenotype related to bone mineralization and homeostasis	Reviewed in [90]

## 4. Channel-Independent Functions of Cx43 on Plasma Membrane

Besides their canonical function as a complete gap junction channel or hemichannel, Cx43 proteins on the plasma membrane also function on other processes. 

### 4.1. Transfer of Mitochondria

As the powerhouse within eukaryotic cells, mitochondria possess a complex double-membrane structure and a size of 0.5 to 3 µm [91]. As a result, mitochondria were thought to be constrained within the plasma membrane for a long time. Recent studies have demonstrated that mitochondria can also be transferred intercellularly, under both physiological and stress conditions, to maintain tissue homeostasis or repair damaged tissue [92].

Several reports have supported a crucial function of Cx43 in the mitochondrial transfer, and the deletion of Cx43 in donor or acceptor cells usually impaired the process [93,94,95]. Given the size of the Cx43 gap junction channel, it is impossible for mitochondria to pass through the channel. Instead, Cx43 is proposed to function as a stabilizer, adhering to the docked membrane structures of two cells [93]. 

A very specialized cellular structure, called tunneling nanotubes (TNT), may participate in intercellular mitochondrial transfer [96,97]. These cell-to-cell contacts, established by long, irregular, thin, moniliform, nanotube-like cytoplasmic processes, express abundant Cx43 at their tips [96]. Mitochondria travel in the TNT, and Cx43 is postulated to form connections between TNTs [96]. However, the mechanisms to remove the cell membrane that separates two cells, to allow exchange of mitochondria, need further investigation. Based on the proposed mechanism, it is not surprising that the content that can be transferred between cells is beyond mitochondria. For example, TNTs induced by HIV infection enable HIV spreading, and Cx43 gap junctions at the ends of their membrane extensions are vital for the process [98].

### 4.2. Regulator of Phagocytosis

A phagosome is a vacuole in the cytoplasm of a cell, containing a phagocytosed particle, enclosed within a part of the cell membrane. Initially, Cx43 was implicated as a regulator of phagocytosis, in response to microbial infection [99]. The inhibition of Cx43, using small interfering RNA or by obtaining macrophages from Cx43 heterozygous or knockout mice, resulted in significantly impaired phagocytosis; meanwhile, transfection of Cx43 into Fc-receptor-expressing HeLa cells, which do not express endogenous Cx43, enabled these cells to undergo phagocytosis upon microbial infection [99].

However, this result was contradicted by a report that found no difference in the phagocytic capabilities between wild type and Cx43 knockout macrophages [100], or by pharmacological blocking of the Cx43 channel, using Gap27 [83]. It is unclear if this lack of difference resulted from compensatory expression of other connexins after the deletion of Cx43, or if the different methodologies and materials that were used impacted the macrophage’s ability to properly perform phagocytosis. Alternatively, the appearance of Cx43 on phagosomes may just be a secondary event of plasma membrane internalization, with Cx43 having no function in the phagocytosis process. 

### 4.3. Cx43 on Extracellular Vesicles

Another membrane structure that Cx43 has been detected on is the extracellular vesicles (EV), although some papers referred to them as the more specific EV population of exosomes [101,102,103,104,105,106]. In fact, exosomes are a subset of extracellular vesicles that have been defined based on their size, biogenesis, and contents; they are used by cells to achieve communication with other cells that are nearby or even at a great distance (reviewed in [107,108]). 

Cx43 is present at the membrane of exosomes as hexamers, forming hemichannels [104]. The presence of Cx43 in exosomes increases the amount of exosomal vesicles detected in target cells, especially when the acceptor cells also express Cx43 [104]. This observation suggests that Cx43 facilitates the docking/fusion of the exosomes with the recipient cell [104], aside from its gap junction function to transmit vesicular contents. It is intriguing that Cx43 extends its role in intercellular communication to a longer distance via exosomes. Biomedical engineering has taken this into improving cytosolic delivery of exosomal contents by overexpressing Cx43 S368A on exosome membranes [109]. 

### 4.4. Is Connexin43 an Active Driver of Membrane Trafficking or a Passenger in the Process?

**Figure 1:**
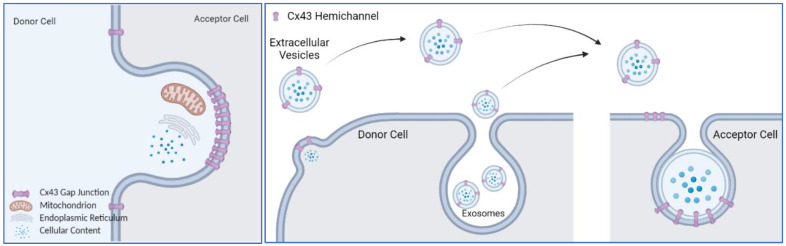
Membrane trafficking with Cx43. (**Left**): Internalization of cellular contents with Cx43 plaque. Cellular contents, including mitochondria, ER and ribosomes can be internalized with Cx43 plaque to form annular Cx43 vesicles. Mitochondria may escape from degradation and carry out function in acceptor cell. (**Right**): Cx43 gap junction mediates internalization of EVs and exosomes. Secreted EVs or exosomes contain functional Cx43 hemichannel on the membrane, that can dock with Cx43 hemichannel on the acceptor cell to make gap junctions and facilitate the internalization (shown here) or fusion of plasma membrane (not shown). Graphs were created with BioRender.com.

A common scheme of the above-listed functions of Cx43 is the dynamic movement of the plasma membrane, either a single layer of plasma membrane or two layers of plasma membranes tethered together. In that case, the question is what role does Cx43 play in this process? Completed gap junctions formed by two Cx43 hemichannels are inseparable under physiological conditions [110,111]; instead, they are removed with the plasma membrane through the internalization process, forming an annular gap junction vesicle or connexosome [112], which is eventually targeted for degradation by autophagosomal pathways [113,114]. Other cellular contents, including mitochondria, can be internalized in the Cx43 gap junction degradation process (Figure 1). It is possible that mitochondrial transfer shares the same pathway of Cx43 gap junction plaque internalization. Then, the internalized mitochondria use a different pathway to escape from lysosomal degradation. If this is the case, it suggests that the Cx43 gap junction internalization machinery could be leveraged to transfer any cellular content that can fit into the internalized vesicle. Phagocytosis can be regarded as an extreme scenario of this phenomena, in which the accepting cell completely internalizes the entirety of the donating cells or foreign objects.

Exosomes are difficult to distinguish from extracellular vesicles by density centrifugation-based purification methods [115]. Secreted vesicles, no matter whether they are exosomes or EVs, contain a hemichannel with an extracellular loop facing the environment, as it is on the plasma membrane [104]. This will allow the EVs to dock with the Cx43 hemichannel on the accepting cell (Figure 1), initiating an internalizing process or fusion of membrane to release their content. 

Other membrane proteins, such as occludins, desmoglein, and desmocollin, can facilitate cell-to-cell interaction, and they usually cluster with gap junction proteins [116]. Both the relative contribution of Cx43 versus other plasma membrane proteins in the membrane tethering, and changes in biophysical properties of the membrane for internalization remain to be investigated. 

## 5. Mitochondria-Localized Connexin43 

Besides the plasma membrane and cytosolic localization, immunostaining of Cx43 has suggested that a pool of Cx43 proteins are located with the mitochondria [117]. Analyzing the Cx43 protein amino acid sequence, using bioinformatic tools, [118] failed to identify a conserved mitochondrial targeting sequence. Recent highly sensitive mitochondrial proteomics studies have usually detected Cx43 in crude mitochondrial fraction, but more stringent criteria, such as enrichment in purified mitochondrial fraction or reduction after mitochondrial import machinery inhibition, usually exclude Cx43 as a mitochondrial protein [119,120]. One caveat for these more stringent criteria is that they may lead to overlooking the possibility of proteins shuttling between locations. 

In 2005, using multiple methods, including immune-gold staining and transmission electron microscopy, Cx43 was demonstrated to be localized to the mitochondria of myocardium and could be further increased by ischemia-reperfusion preconditioning (IPC) in isolated rat hearts and pig myocardium [121]. The heart is extremely energetic, heavily relying on mitochondria for supplying energy. Ischemia-reperfusion injury is the tissue damage caused by blood supply returning to tissue (reperfusion) after a period of ischemia or lack of oxygen (anoxia or hypoxia) [122]. During ischemia, the diminished oxygen level has a direct impact on mitochondria respiration and their function, making them an important target for cardioprotection [123,124]. The IPC of myocardium is a well-described adaptive response, in which brief exposure to ischemia-reperfusion takes place before sustained ischemia. IPC markedly enhances the ability of the heart to withstand a subsequent ischemic insult [122,125]. Therefore, there is great interest in understanding the role of mitochondria in preconditioning. 

Cx43 is encoded by the nuclear genome, and the rapid increase in Cx43 in mitochondria, in response to IPC, is achieved by shuffling cytosolic Cx43 to the mitochondria via heat shock protein 90 (Hsp90) [126]. Once Cx43 reaches the outer mitochondrial membrane, it is imported through the translocase of the outer membrane (TOM) complex and subsequently inserted into the inner mitochondrial membrane through the translocase of the inner membrane complex (TIM) [126]. Intriguingly, cardiomyocytes contain the following two mitochondrial subpopulations: the subsarcolemmal (SSM) and the interfibrillar (IFM) mitochondria, which have different morphology and functions [127]. SSM shows a reduced oxidative phosphorylation rate compared to IFM, as well as increased resistance to various stress stimuli [127]. Mitochondria-localized Cx43 (mtCx43) is almost exclusively present in SSM [128]. Proteinase K digestion of isolated cardiomyocyte SSM mitochondria suggests that the C-terminal of mtCx43 is toward the inter-membrane space [128], which was later confirmed by another report, using immunofluorescence against the C-terminus domain of Cx43, under different mitochondrial permeabilization methods [129]. Since then, the majority of the mtCx43 studies have been carried out in cardiomyocytes, mostly in the context of IPC. 

Despite a clear protective role in IPC, the molecular function of mtCx43 remains to be elucidated. Experiments have supported that mtCx43 oligomerizes and forms hemichannels on the mitochondrial inner membrane [129]. The absence or pharmacological blockade of mtCx43 reduces dye and potassium uptake, suggesting that the mtCx43 hemichannel on the mitochondrial inner membrane is functional [130]. Assuming a similar gating mechanism of the mtCx43 hemichannel to the plasma membrane hemichannel, +60 mV potential is needed to activate the hemichannel, which implies that mtCx43 would be constantly open, as the electrical potential across the mitochondrial inner membrane is 150–180 mV. However, this scenario is unlikely to happen, as it would collapse any ion gradient segregated by the inner mitochondrial membrane. It would be interesting to test whether mitochondria-targeted overexpression of Cx43-A44V or Cx43-E227D, two Cx43 mutants with much higher hemichannel conductance [131], would collapse mitochondrial potential and lead to mitochondrial dysfunction. 

Nevertheless, mtCx43 has been postulated in mitochondrial potassium uptake. The negative charge of the mitochondrial matrix creates an electrical gradient that drives K^+^ entry into the mitochondrial matrix, despite the similar concentrations of this cation in mitochondria and the cytoplasm [132]. Potassium import maintains the structural integrity of the mitochondria [133] while increasing mitochondrial respiration and the generation of reactive oxygen species (ROS) [132]. During ischemia-reperfusion injury, increased mtCx43, as well as increased S-nitrosation of mtCx43, boost the mitochondrial permeability for potassium and lead to increased ROS formation [130], which activates signal cascades that are critical for the cardiac protection of IRI [134]. A lack of mtCx43 leads to the loss of formation of ROS upon IPC and is associated with a loss of endogenous cardioprotection by IPC [130]. 

One rationalized function of mtCx43 would be the regulation of mitochondrial respiration. Given the permeability of the Cx43 hemichannel to protons and the existence of a proton gradient across the mitochondrial inner membrane, one would suspect that mtCx43 may uncouple mitochondria. One study of the Cx43 gap junction channel showed a bell-shaped pH dependence of proton permeability curve, with the peak of proton conductance at a pH of 7.2 [135]. If similar regulation of proton permeability between the hemichannel and the complete channel can be assumed, mtCx43 hemichannel should remain permeable to protons, given that the mitochondrial interspace has a pH value of 7.0. However, an additional mechanism to shut down the channel in a timely fashion must exist to maintain a proton gradient across the mitochondrial inner membrane. This mechanism is still waiting to be discovered. 

Cellular energy measurements showed that Cx43-overexpressing HL-1 cardiomyocytes complex I respiration was increased, whereas complex II respiration remained unaffected; in contrast, inhibition of Cx43 in a rat’s left ventricular mitochondria also reduced ADP-stimulated complex I respiration and ATP generation [136]. Whether mtCx43 has a direct effect on Complex I respiration, or the increase in Complex I respiration is a secondary effect of mtCx43-mediated mitochondrial uncoupling that results in higher demand of electron transport remains to be studied. 

A few studies have claimed the mitochondrial localization of Cx43 in other cell types, i.e., brown adipocytes [137] or retinal endothelial cells [138,139]. Experimentally, there have been historical constraints raised by traditional biochemistry dogma. Mitochondrial preparation is inevitably contaminated by ER proteins, and fluorescent imaging without 3D reconstruction may show signal overlay from different Z-planes or rare artifacts of colocalization. Many follow-up studies fell into the pitfalls of these constraints. A mitochondrial localization of Cx43 in other types of cells should not be taken for granted because, in cardiomyocytes, only SSM mitochondria have Cx43. IFM mitochondria in the same cardiomyocytes do not have Cx43. Thus, experimental rigor is required to definitively show the subcellular localization and function of mtCx43 inside of those non-cardiomyocytes.

**Figure 2:**
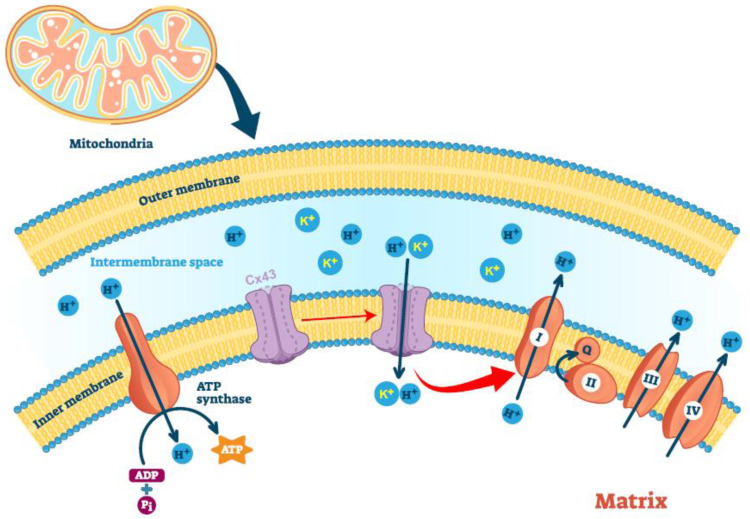
Mitochondria-localized Connexin43 forms a function hemichannel and may regulate potassium uptake, mitochondrial uncoupling, and ROS production. Cx43 forms a function hemichannel on mitochondrial inner membrane with C-terminal tail facing the intermembrane space. The mtCx43 channel is expected to let potassium and protons go through in a regulated way, which would in turn regulate mitochondrial integrity, Complex I respiration and ROS generation.

In summary, many aspects of mtCx43 have been worked out (Figure 2). However, many questions remain to be answered. For example, what determines the Cx43’s migration into the SSM but not the IFM mitochondria in cardiomyocytes? Other types of cells do not have clear subpopulations of mitochondria in them; will Cx43 appear in the only kind of mitochondria in those cells? And what is the driving force of the mitochondrial localization of Cx43 without a mitochondrial targeting sequence? Mouse models with cardiac-specific overexpression of mtCx43 have not been established, after many years following the discovery of the beneficial functions of mtCx43 in IPC. This mouse model will definitely answer many questions and would be an important tool to study mtCx43. 

## 6. Alternative Translation of Connexin43 mRNA 

Immunoblotting against Cx43 using antibodies usually rendered multiple bands below the predicted molecular weight of 43 kD [140]. A careful examination excluded the possibility of Cx43 protein degradation during sample preparation for western blotting, instead, demonstrating that those bands were bona fide translated polypeptides, corresponding to C-terminal fragments of the Cx43 protein [140]. However, how those Cx43 C-terminal fragments formed and whether they had a biological function was not clear for years, until a seminal report demonstrated that GJA1 mRNA could undergo an alternative translation initiation event to generate several shorter Cx43 protein fragments in cardiomyocytes [141]. One of the most abundant truncated protein isoforms, with a predicted molecular weight of 18.5 kD, was termed GJA1-20k [141]. Given that the name is for a protein, we hereafter refer to it as Cx43-20k. Mouse, rat, and human Cx43 protein all have 382 amino acids, with >97% amino acids identical between species. All internal ATG codons (coding methionine) localize at the exact same location, including the ATG codon for Cx43-20k, which corresponds to the Cx43 amino acid sequence of 213 to 382.

Canonical protein translation uses a cap-dependent mechanism. The process involves the recruitment of various eukaryotic initiation factors at the mRNA 5′-untranslated region (5′-UTR) and the binding of ribosomes to form an initiation complex. Once recruited, this initiation complex starts to scan the transcript from the 5′-UTR to locate the AUG start codon and initiate translation [142]. The recognition of the AUG triplet as a translation initiation signal depends on its nucleotide context, such as its Kozak sequence [143]. However, alternative translation mechanisms are also utilized for mRNA molecules. For example, internal ribosomal entry sites, secondary structures, or upstream open reading frames in the 5′ UTR region usually lead to the usage of alternative start codons, resulting in alternative protein isoforms [144,145]. The 5′-UTR of Cx43 mRNA, which contains 208 nucleotides, has a stable secondary structure, revealed by computer-aided folding models, which can be inhibitory to the scanning of the 40S ribosome [146]. Experimental analysis revealed a functional internal ribosome entry site toward the 5′ end of the Cx43 mRNA UTR [146]. However, this would not explain why Cx43 mRNA translates much smaller C-terminal fragments. Later, another report found a putative IRES element present in the coding region of Cx43, and it was enhanced during chemically induced ischemic deprivation in astrocytes and in ischemic brains [147]. However, truncation studies demonstrated that 5′ cap and upstream ribosome scanning is still required for translation of Cx43-20k [141], arguing against the importance of an internal ribosome entry site in the GJA1 mRNA 5′ UTR or within the coding region of Cx43 [146,147]. Instead, the authors proposed a “leaky scanning” theory [148], suggesting that the 5′ UTR nucleotide context is suboptimal, so a portion of 40S ribosomal subunits skip it, continuing to scan in the 3′-direction and initiating translation at downstream AUGs. 

What adds even more complexity to this research are the dynamic alterations in transcription start sites yielding many *Gja1* 5′ UTR variants, differing only in 5′ UTR length [149]. These could contribute to differences in translation efficiency and alternative translations. However, a detailed molecular mechanism leading to the translation of Cx43-20k still remains to be elucidated.

In cardiomyocytes, levels of Cx43-20k are dynamically regulated by growth factor signaling and cellular stress, such as hypoxia [149]. Several molecular functions of Cx43-20k have been identified. Cx43-20k binds to microtubules, regulating trafficking of full-length Cx43 to the plasma membrane [150]. The ectopic expression of Cx43-20k protects against Cx43 gap junction loss during acute ischemia [151]. The deletion of Cx43-20k by mutating the internal AUG led to the impairment of Cx43 moving to the plasma membrane and the degradation of poorly trafficked Cx43, suggesting a critical role of Cx43-20k in Cx43 gap junction trafficking, the maintenance of Cx43 protein, and the normal electrical function of the mammalian heart [152]. Authors also carefully examined Cx43 stability and found this point mutation did not affect Cx43 protein per se [152]. Cx43-20k also regulates the transport of mitochondria to mediate cellular response to oxidative stress, preserving mitochondrial localization and function [153]. Under cellular stress, Cx43-20k facilitates polymerization of actin around mitochondria to form focal constriction sites to initiate mitochondrial fission [154].

In summary, Cx43-20k and other isoforms, derived from the same Cx43 mRNA, demonstrate the adaptation of a single mRNA to produce several proteins that perform multiple independent or related functions. This greatly facilitates the functional plasticity of the proteome, allowing a limited number of protein-coding genes to perform a multitude of cellular processes.

## 7. Cx43 C-Terminal Fragment Generated by Proteomic Cleavage

Cx43-20K possesses a transmembrane domain and functions in cytosol [150,151,152,153,154]. Smaller fragments without any transmembrane domain, collectively called Cx43 C-terminal fragments (Cx43-CT), have been suspected to exist, and some of them have a unique nuclear localization [155,156]. This may explain previous observations of the nuclear signal of Cx43 immuno-staining, given that the antibody used recognizes the C-terminal of the Cx43 protein [157]. Many nucleus-localized proteins possess short stretches of amino acids, termed nuclear localization signal peptides, which interact with the nuclear pore complex to facilitate the active translocation of the protein into the nucleus [158]. Bioinformatical inquiry against a nuclear localization signal database NLSdb [159] shows no consensus regarding the nuclear targeting sequence in the C-terminal cytoplasmic tail of the Cx43 protein, despite strong experimental evidence supporting the nuclear enrichment of Cx43-CT [155,156]. 

Despite their existence, the production of transmembrane-free Cx43-CT is largely unsolved. One report hints that intracellular MMPs, specifically MMP7, may play a role [160]. Active MMP-7 dose-dependently reduced the Cx43 full-length band of recombinant Cx43 protein and left ventricle extracts in test tubes [160]. The cleavage of Cx43 by MMPs not only renders small, free endogenous C-terminal peptides that are potentially bioactive with physiological functions, it also results in a truncated Cx43 protein without a C-terminal cytosolic tail [160]. However, a corresponding dose-dependent increase in predicted fragments was not observed in the report [160].

MMP7 belongs to a large matrix metalloproteinases family of calcium-dependent, zinc-containing endopeptidases that are capable of degrading extracellular matrix proteins and bioactive molecules [161]. However, the enzymatic activity of many MMPs is not confined to the extracellular space, and substrates are much more diverse than initially thought. At present, the intracellular matrix proteins themselves, as well as their enzymes and molecular chaperones, are well known to be substrates of multiple MMPs [162]. A silico analysis, using PROSPER and SitePrediction, has revealed more than a dozen potential cleavage sites of MMP-2, MMP-7, and MMP-9 in the human Cx43 C-terminal domain [163]. The experimental evidence of MMP7’s intracellular activity has been previously reported as well [164,165,166], and the binding of Cx43 to MMP7 was confirmed in vitro [160]. This all supports a potential role of MMP7 in the proteolytic cleavage of Cx43 and generating Cx43-CT. 

Based on the bioinformatic prediction of several potential DNA-binding motifs in the sequences of Cx43 [167] and the observation of cell growth inhibition effects of overexpressed Cx43-CT [155,156], one suspected function of these nuclear Cx43-CT proteins is mediating gene expression. A recent report shows that a nuclear Cx43 fragment interacts with basic transcription factor-3 (BTF3) to form a complex with PolII that directly binds to the N-cadherin promoter to regulate its transcription in the *Xenopus cephalic* neural crest cell in vivo [168]. It is worth noting that the identity of the endogenous Cx43 fragment remains obscure in this paper, and whether the use of GFP fusion protein artificially drives the nuclear localization of the Cx43 C-terminal fragment remains a concern, as GFP has been shown to specifically direct some proteins to the nucleus, without the involvement of any NLS peptides [169].

In summary, the Cx43-CT protein that corresponds to amino acids 244 to 382 of the Cx43 protein shows nuclear localization and growth inhibition in cell cultures. Whether this can be endogenously produced and whether these effects can translate into animal models is still unclear and requires more work.

## 8. Summary and Future Directions

Cx43 uses multipronged mechanisms to expand its functional protein pool from just one gene, vividly demonstrating how sophisticated a biological system can be. According to the most recent consensus coding sequence (CCDS) project releases, the number of mouse genes has been significantly lowered over the years from an initial estimate of 30,000 to just over 20,000 [170,171]. However, more mRNA isoforms are generated by alternative splicing. For example, there are more than 75,000 mRNA isoforms encoded by over 20,000 genes in the mouse genome annotation (GRCm38.p4) [172,173]. Additionally, the alternative translation initiation [174], post-transitional modification [175], and cleavage [176] of the protein further expands the functional protein pool.

With the core function of connecting two cells together, the full-length Cx43 protein’s function expands to paracrine signaling through its hemichannel activity and the regulation of mitochondrial dynamics and function. Many additional protein products can be derived through mRNA and protein processing. These proteins also each have cellular functions that are both related and unrelated to Cx43. As such, the questions are as follows: Is Cx43 unique in the connexin family in possessing all of these regulations? Or are these observations just a reflection of the fact that Cx43 has been the most extensively studied? Given a big family of connexin genes, a lot of work remains to be done on other connexins. Even for Cx43, many aspects of its biology remain to be discovered. For example, Cx43 mRNA can be translated to several additional polypeptides besides Cx43-20k, and the functions of those alternative translated Cx43 isoforms remain unknown. The underlying molecular mechanism of mtCx43 and Cx43-CT function also remains to be illustrated. 

One very interesting observation is that Cx43 is probably the most toxic connexin in the family [177], enhancing apoptosis [178] or significantly inhibiting cell proliferation [179,180,181]. Where does this toxicity come from? This question may even be asked in a philosophical manner, as follows: What is the developmental origin of the *Gja1* gene? How did all of the different aspects of the Cx43 process evolve at the molecular level, and how did those different peptides gain very distinct functions? 

Because the Cx43 mutation-related diseases are mostly congenital, involving early development, these diseases are extremely difficult, if not impossible, to treat. To the author’s knowledge, no effective medication has been approved that specifically targets Cx43 for those congenital diseases [17]. Special considerations for a housekeeping role of Cx43 and many other connexin isoforms with similar structures, expressed in various tissues, are being explored in order to develop Cx43-based therapy. Currently, the most explored therapeutic area of Cx43-based therapy is on skin diseases [17]. However, leveraging the Cx43 gap junction, either for “bystander killing” to treat cancer, or the “good Samaritan” effect, to enhance a desired beneficial signal, are exciting concepts that are being actively pursued. New concepts of using Cx43 as a tool to develop the specific liposomes that need to be delivered to the Cx43 expression cells are also being tested [105]. Among many new strategies, connexin peptide mimetics have demonstrated their usefulness, and biochemical modifications have greatly improved its isoform specificity, tissue specificity, cell membrane penetrance, and half-life [182]. After six decades of research on Cx43 and connexins, there is still a lot of Cx43 biology to explore. Hopefully, we will be able to translate these years of discovery and knowledge accumulation into a therapy for patients in the future.

## Data Availability

Not applicable.
